# Resveratrol and cancer: focus on *in vivo* evidence

**DOI:** 10.1530/ERC-13-0171

**Published:** 2014-06

**Authors:** Lindsay G Carter, John A D'Orazio, Kevin J Pearson

**Affiliations:** 1 Graduate Center for Nutritional Sciences, Markey Cancer Center, University of Kentucky College of MedicineWethington Building, Room 591, 900 South Limestone, Lexington, Kentucky, 40536-0200USA; 1 Department of PediatricsGraduate Center for Toxicology, Markey Cancer Center, University of Kentucky College of MedicineLexington, Kentucky, 40536-0096USA

**Keywords:** colon, mammary gland, obesity, phytoestrogen, prostate

## Abstract

Resveratrol is a naturally occurring polyphenol that provides a number of anti-aging health benefits including improved metabolism, cardioprotection, and cancer prevention. Much of the work on resveratrol and cancer comes from *in vitro* studies looking at resveratrol actions on cancer cells and pathways. There are, however, comparatively fewer studies that have investigated resveratrol treatment and cancer outcomes *in vivo*, perhaps limited by its poor bioavailability when taken orally. Although research in cell culture has shown promising and positive effects of resveratrol, evidence from rodents and humans is inconsistent. This review highlights the *in vivo* effects of resveratrol treatment on breast, colorectal, liver, pancreatic, and prostate cancers. Resveratrol supplementation in animal models of cancer has shown positive, neutral as well as negative outcomes depending on resveratrol route of administration, dose, tumor model, species, and other factors. Within a specific cancer type, there is variability between studies with respect to strain, age, and sex of animal used, timing and method of resveratrol supplementation, and dose of resveratrol used to study cancer endpoints. Together, the data suggest that many factors need to be considered before resveratrol can be used for human cancer prevention or therapy.

## Introduction

Resveratrol (*trans*-3,5,4′-trihydroxystilbene) is a phytoalexin found in many plant species, including those often consumed by humans such as grapes, peanuts, and berries; it is produced in plants in response to mechanical injury, fungal infection, and u.v. radiation (Langcake & Pryce 1976). The highest naturally occurring levels of resveratrol are found in *Polygonum cuspidatum* (Japanese knotweed), a plant which has been used for hundreds of years in traditional Asian medicine to treat inflammation and other ailments (Vastano *et al*. 2000, Burns *et al*. 2002). Concentrations of resveratrol vary markedly between plant species. In blueberries, for example, resveratrol concentrations approximate only 32 ng/g, compared with levels up to 1920 and 3540 ng/g in peanuts and grapes respectively (other beneficial compounds are also present in varying quantities; Sanders *et al*. 2000, Burns *et al*. 2002, Lyons *et al*. 2003). Resveratrol is not only found in these plants, but also in processed products such as wine. In fact, many attribute the ‘French Paradox’ in which moderate wine consumption is associated with decreased risk of coronary heart disease (Renaud & de Lorgeril 1992), to be the result of red wine's relatively high resveratrol concentration (0.1–14.3 mg/l) (Goldberg *et al*. 1995, Kiraly-Veghely *et al*. 1998, Kopp 1998, Pervaiz 2003). Nonetheless, wine's resveratrol content is typically much lower than what has been shown experimentally to have health benefits, but recent work has suggested that lower levels of resveratrol can also provide health improvements (Tome-Carneiro *et al*. 2012). For a review and discussion of the clinical literature along with the limitations of preclinical and *in vitro* resveratrol studies, see Tome-Carneiro *et al*. (2013). Further, Baur & Sinclair (2006) provide a thorough review of the *in vivo* effects of resveratrol on many disease states.

Because resveratrol is a naturally occurring compound, it has been highly studied for the prevention and treatment of many diseases including cancer. After Jang *et al*. (1997) found that topical application of resveratrol protected mice from tumorigenesis in a skin cancer model in 1997, a wealth of publications followed. In animals, supplemental doses of resveratrol protect against many of the deleterious effects of high-fat diets and provide additional health benefits (Hung *et al*. 2000, Bradamante *et al*. 2004, Baur *et al*. 2006, Lagouge *et al*. 2006, Pearson *et al*. 2008, Ramadori *et al*. 2009, Kang *et al*. 2010). Numerous *in vitro* studies have shown that resveratrol has multiple anti-cancer effects, protecting against both tumor initiation and cancer progression pathways. For example, resveratrol can promote cell cycle arrest leading to apoptosis of tumor cells, prevent tumor-derived nitric oxide synthase expression to block tumor growth and migration, as well as act as an antioxidant to prevent DNA damage that can lead to tumor formation (Clement *et al*. 1998, Tsai *et al*. 1999, Nakagawa *et al*. 2001, Murakami *et al*. 2003, Garvin *et al*. 2006, Kalra *et al*. 2008). In addition, resveratrol inhibits cyclooxygenase (COX) activity, which is known to play a role in tumorigenesis by converting arachidonic acid to prostaglandins, inflammatory compounds that promote tumor cell proliferation (Subbaramaiah *et al*. 1998, Jang & Pezzuto 1999, MacCarrone *et al*. 1999). Resveratrol has also been shown in multiple studies to decrease DNA binding activity of nuclear factor κB (NF-κB), which is a transcription factor that is known to be upregulated in cancers and can drive the transcription of genes that promote tumor growth (Holmes-McNary & Baldwin 2000, Benitez *et al*. 2009, Csaki *et al*. 2009, Roy *et al*. 2009).

Resveratrol appears to have many anti-tumor effects on different cancer cells *in vitro* and these effects and pathways have been extensively reviewed (Bhat & Pezzuto 2002, Dong 2003, Le Corre *et al*. 2005, Kundu & Surh 2008, Shukla & Singh 2011). Regarding *in vivo* evidence, Jang *et al*. (1997) were the first to show that resveratrol may act as a chemopreventative agent when they found that topical application of the compound was able to inhibit tumor formation in the two-stage skin cancer model in mice. Later studies found that in mouse models of skin tumorigenesis, topical resveratrol prevented tumor formation by promoting apoptosis, regulating the cell cycle, and inhibiting COX activity and prostaglandin production (Afaq *et al*. 2003, Reagan-Shaw *et al*. 2004, Kalra *et al*. 2008). The *in vivo* use and efficacy of resveratrol for other types of cancer that require oral consumption or injection of resveratrol, however, have been less straight forward. This is due, in part, to the poor bioavailability of *trans*-resveratrol. Wenzel & Somoza (2005) provide a critical and detailed review of the bioavailability and metabolism of resveratrol. In rodents and humans, when resveratrol is consumed orally, 70–80% is quickly absorbed via passive diffusion in the intestines (Andlauer *et al*. 2000, Soleas *et al*. 2001, Kaldas *et al*. 2003, Walle *et al*. 2004). After absorption, resveratrol is conjugated into glucuronides and sulfates, so that circulating levels of *trans*-resveratrol peak 30–60 min post oral administration (Andlauer *et al*. 2000, De Santi *et al*. 2000, Soleas *et al*. 2001, Yu *et al*. 2002). In humans, circulating levels of unmodified *trans*-resveratrol are only ∼2% of the peak serum concentration of total free resveratrol and conjugates after a single dose of 25 mg/70 kg body weight (BW; Goldberg *et al*. 2003). Another report shows that at least 70% of resveratrol is absorbed after a single 25 mg dose, and there is a peak serum concentration of 2 μM (∼490 ng/ml) for resveratrol and all of its metabolites (Walle *et al*. 2004). After multiple oral doses (5 g daily for 29 days), plasma concentrations of *trans*-resveratrol have been reported to be as high as ∼4 μM (4.29 nmol/ml); however, it should be noted that resveratrol at this high dose was also associated with gastrointestinal side effects (Brown *et al*. 2010). Interestingly, in human colon tissue, levels of resveratrol and its metabolite resveratrol-3-*O*-glucuronide have been found at high concentrations (674 and 86 nmol/g respectively) when 0.5–1.0 g of resveratrol was taken orally once per day (Patel *et al*. 2010). In this study, resveratrol supplementation was shown to decrease cellular proliferation by 5% in colorectal cancer tissue, as assessed by Ki67 staining (Patel *et al*. 2010). Since there is such rapid conjugation and low bioavailability of resveratrol, the *in vivo* use of resveratrol for cancer prevention and treatment is uncertain. Therefore, it is the intention of this review to highlight findings from *in vivo* studies.

First, this review will briefly discuss the limited clinical evidence currently available on resveratrol and cancer treatment and prevention. Then, given the vast amount of research done with resveratrol and cancer (a PubMed search of ‘resveratrol and cancer’ yielded more than 1800 hits) and the more recent interest in obesity as a risk factor for cancer, this review will focus primarily on the *in vivo* studies involving resveratrol and several obesity-related cancers; specifically breast, colorectal, hepatic, pancreatic, and prostate cancers. Tables 1, 2, 3, 4 and 5 summarize the methods and outcomes of *in vivo* experiments that have tumor formation as an endpoint measurement rather than those studies that investigate the mechanisms, biomarkers, or pathway changes.

## Clinical studies

Clinical evidence for resveratrol as an effective supplement for cancer prevention and treatment is scarce. In 2009, the first phase I clinical trial looking at resveratrol treatment in patients diagnosed with cancer was published (Nguyen *et al*. 2009). Patients with colorectal cancer (*n*=8) had normal and cancerous intestinal mucosal samples biopsied at the time of diagnosis and 14 days after daily resveratrol (20 or 80 mg/day; *n*=2 and 1 respectively) or grape powder (80 or 120 g/day; *n*=3 and 2 respectively) oral supplementation at the time of colon cancer resection surgery. The Wnt signaling pathway, known to be involved in the formation of colon cancer, was evaluated in normal and cancerous mucosa, before and after resveratrol or grape powder supplementation. Target genes in the Wnt pathway were significantly higher in cancerous compared with normal mucosa. Resveratrol and grape powder administration had no effect on cancerous mucosa Wnt signaling, but their supplementation resulted in decreased Wnt target gene expression in normal mucosa (effects of all treatment groups combined). The most significant effects were observed with the low-dose grape powder. This led the authors to conclude that resveratrol in combination with other compounds found in grapes could possibly be used to decrease the risk of colon cancer development by decreasing Wnt pathway signaling, but might not be as effective against established colon cancer. The second clinical study observed the effects of resveratrol treatment in colorectal cancer patients with hepatic metastasis (*n*=9). Resveratrol supplementation (5 g daily of microionized resveratrol SRT501 for 10–21 days; *n*=6) increased the expression of cleaved caspase-3 in cancerous hepatic tissue, indicating increased apoptosis of cancerous cells compared with those of placebo-treated subjects (*n*=3) (Howells *et al*. 2011). It is important to caution that patient sample size in these clinical trials was small (only eight and nine cancer patients were enrolled in the studies respectively), highlighting the fact that so far, there is very little human data for the efficacy of resveratrol in cancer treatment.

A few other clinical studies have focused on resveratrol supplementation and predictors for cancer prevention and cancer risk factors in healthy subjects. Given that increases in insulin-like growth factor 1 (IGF1) and decreases in IGF-binding protein 3 (IGFBP3) are associated with tumor formation and metastasis, one study looked at the effects of resveratrol supplementation (0.5, 1.0, 2.5, and 5 g/day for 29 days; *n*=10–12/dose) on circulating levels of these proteins (Brown *et al*. 2010). After 29 days of supplementation, the authors found that resveratrol treatment at 2.5 g/day significantly reduced IGF1 and IGFBP3 levels in plasma, which would support the use of resveratrol as a chemopreventative agent in humans. The 1.0 g/day dose also caused a significant decrease in plasma IGFBP3 compared with pretreatment baseline levels. The two higher doses did cause some short-term mild to moderate gastrointestinal symptoms in multiple subjects (Brown *et al*. 2010). In another trial, healthy subjects were given 1 g of resveratrol for 4 weeks and lymphocyte levels or surrogate markers of activity levels of enzymes involved in carcinogenesis and drug metabolism were measured (Chow *et al*. 2010). Resveratrol supplementation increased the protein or activity levels of a variety of carcinogen-detoxifying enzymes, such as glutathione *S*-transferase and glucuronosyltransferase, but a significant increase was only reached where enzyme levels were low at baseline. Chow *et al*. (2010) noted the important caveat that although pharmacologic resveratrol supplementation seems well tolerated and may exert a cancer-protective effect through enhanced detoxification of carcinogens, it might also have the potential to alter the metabolism of a variety of medications through the inhibition of cytochrome P450 activity. Therefore resveratrol's safety and benefit must be further delineated, particularly in the context of co-administering it with pharmaceutical agents.

From this limited clinical trial data, it is apparent that much more human research is needed before resveratrol can be considered as a viable option for cancer prevention or therapy. There are several other completed clinical trials looking at resveratrol and cancer that have yet to publish results and one on-going clinical trial (clinicaltrials.gov). All of these trials are focusing either on patients with colorectal cancer or are assessing cancer prevention capabilities of resveratrol in healthy patients. Thus far, its most promising use seems to be in cancer prevention instead of treatment. It is important to note that there is some evidence that resveratrol may have adverse effects in certain cancer patients. In a phase II clinical trial involving relapsed or refractory multiple myeloma patients, resveratrol at a dose of 5 g/day caused adverse events (including nausea, diarrhea, fatigue, and renal toxicity), which may have contributed to the death of one patient and caused the investigators to prematurely end the study (Popat *et al*. 2013). The authors note that this high dose has been shown to be safe in other clinical trials in healthy patients. This highlights the need for more research into the efficacy and safety of resveratrol in *in vivo* cancer models. Later we will discuss much of the work that has been completed with resveratrol use in animal models of breast, colorectal, hepatic, pancreatic, and prostate cancers.

## Animal studies

### Breast cancer

Breast cancer accounts for one in three diagnosed cancers in women in the USA (DeSantis *et al*. 2011*a*,b). Current treatment options for breast cancer include chemotherapy, radiation, or surgery to remove tumors and breast tissue. Hormone therapy is also available, especially for post-menopausal women. Resveratrol is considered a phytoestrogen that seems to have both agonistic and antagonistic effects on estrogen (Bowers *et al*. 2000, Bhat *et al*. 2001). Given this, it makes sense that research conducted on resveratrol and estrogen-related cancers have found diverse results. In several animal models, resveratrol supplementation was shown to decrease the incidence of mammary tumor formation. In 45-day old female Sprague–Dawley rats, resveratrol supplementation in the diet (0.001%; daily intake calculated to be 100 μg/rat) started 7 days before tumor initiation and continued for 120 days after initiation was found to increase time to first tumor formation and decrease tumor incidence and multiplicity following 7,12-dimethylbenz(*a*)anthracene (DMBA) administration (Banerjee *et al*. 2002). Analysis of the tumor tissue also showed that resveratrol reduced DMBA-generated *COX2* expression and NF-κB binding to DNA. In a similar rat mammary tumor model, supplementation of a higher dose of resveratrol in the diet (0.1%), starting at birth and continuing for 180 days, decreased tumor number per rat and increased latency to tumor development after tumor initiation at postnatal day 50 (Whitsett *et al*. 2006). For resveratrol supplementation starting at birth, nursing dams were fed the diets containing resveratrol and pups were then weaned onto the same diet. Cellular proliferation and apoptosis in the mammary tumor tissue was also measured. Proliferating cell percentage was reduced with resveratrol treatment, while apoptotic-labeling index (epithelial cells stained positive for apoptosis/total number of epithelial cells) was increased compared with control diet-fed rats (Whitsett *et al*. 2006).

Using young, 5-week-old female Sprague–Dawley rats and a DMBA carcinogenesis model, Chatterjee *et al*. (2011) found that resveratrol supplementation in the diet (0.001%; daily intake calculated to be 100 μg/rat) decreased palpable mammary tumor incidence 11 weeks after DMBA exposure. After 24 weeks of supplementation, animals were killed and mammary tissue was analyzed for DNA damage, 5-lipoxygenase (5-LOX), transforming growth factor β1 (TGFβ1), NF-κB, cell proliferation, and apoptosis, all of which can be the indicators of tumorigenesis. Resveratrol treatment positively impacted all of these markers in the mammary tissue; it decreased the appearance of single-strand DNA, indicating less DNA damage; decreased 5-LOX expression and activity; decreased TGFβ1 and NF-κB expression; decreased cell proliferation; and increased the number of apoptotic cells. In a different model of rat mammary tumorigenesis using *N*-nitroso-*N*-methylurea (NMU) to promote tumor formation in 49-day-old Sprague–Dawley females, resveratrol was given by oral gavage (10 or 100 mg/kg BW) five times a week for 1 week before NMU injection and continued for 120 days after. The higher dose of resveratrol resulted in a significant delay in tumor formation and decrease in tumor multiplicity, while the lower dose did not significantly alter these parameters compared with control (Bhat *et al*. 2001). In a model of spontaneous mammary tumor formation, 20-week-old female FVB/N HER2/*neu* transgenic mice treated with resveratrol in their drinking water (0.0001%; daily intake calculated to be 4 μg/mouse) for 11 weeks had a significant increase in latency to tumor formation. Resveratrol treatment also decreased tumor number and size per animal and reduced tumor metastasis to the lungs (Provinciali *et al*. 2005). In a xenograft model where athymic nude female mice from 6 to 8 weeks of age were injected with MDA-MB-231 (estrogen receptor (ER)α(−), ERβ(+)) cells, Garvin *et al*. found that resveratrol (25 mg/kg BW; i.p. injection) given daily for 3 weeks after tumors had already reached 40 mm^3^ caused a significant reduction in tumor growth. Tumors in vehicle-treated controls increased in size by four- to fivefold over the next 3 weeks, while tumors in resveratrol-treated mice did not increase in size. Furthermore, apoptosis was increased and angiogenesis was decreased in the tumor cells from resveratrol-compared with vehicle-treated mice (Garvin *et al*. 2006). Taken together, these animal models suggest that resveratrol could potentially be used as a chemopreventative or cancer treatment agent.

Other studies have not shown such promising results for resveratrol as a breast cancer therapy. In 17-week-old BALB/c female mice, 4T1 mammary carcinoma cells (an ERα- and Erβ-positive cell line) were injected and breast tumor formation and metastasis to the lungs were monitored (Bove *et al*. 2002). Mice that were treated with resveratrol at 1, 3, or 5 mg/kg BW daily (i.p. injection) for 23 days following tumor cell injection showed no differences in mammary tumor latency, mammary tumor number, or tumor metastasis to the lungs compared with vehicle-treated mice injected with the 4T1 cells (Bove *et al*. 2002). Castillo-Pichardo *et al*. injected low metastatic cells ERα(−), ERβ(+) MDA-MB-231, or high metastatic ER(−) MDA-MB-435 cancer cells into 5- to 6-week-old female mice with severe combined immunodeficiency (SCID) and athymic nude (nu/nu) respectively. They then evaluated tumor formation and metastasis with or without resveratrol supplementation (0.5, 5, or 50 mg/kg BW; oral gavage) starting 7 days after tumor cell injection and continuing for 44 or 108 days (Castillo-Pichardo *et al*. 2013). At all concentrations of dietary supplementation and in both cell types, there was an increase in mammary tumor formation and metastasis compared with vehicle-treated mice. Because phytoestrogens such as resveratrol can affect development in prepubertal animals, Sato *et al*. (2003) treated female Sprague–Dawley rats daily with resveratrol on postnatal days 15–19 (10 or 100 mg/kg BW; s.c. injection). On postnatal day 49, rats were injected with NMU to promote mammary tumor formation. Resveratrol at 100 mg/kg BW given on postnatal days 15–19 had no effect on tumor latency but increased the multiplicity of tumors and the incidence of rats with tumors ≥1 cm. The lower dose did not cause the same negative effects, but it was not beneficial either (Sato *et al*. 2003). These studies suggest that caution must be applied in adapting resveratrol for human use and may indicate that resveratrol can promote mammary tumor growth and formation depending on cell type and other factors. A summary of these studies is provided in Table 1.

### Colorectal cancer

Colorectal cancer is one of the leading causes of cancer deaths in the Western world (Siegel *et al*. 2012). Diet and lifestyle have been shown to have a significant impact on the development of colorectal cancers (Doll & Peto 1981, Willett 1995), making resveratrol an interesting treatment possibility for this cancer type. Furthermore, oral administration of resveratrol might be expected to have a maximal impact on local intestinal processes before metabolic inactivation by the liver. Several different animal models have been used to evaluate the effect of resveratrol treatment on colon tumor formation. Using azoxymethane to induce colon tumorigenesis in 8-week-old male Fisher 344 rats, Tessitore *et al*. (2000) found that resveratrol supplementation in drinking water (daily intake calculated to be 200 μg/kg BW) for 100 days decreased the appearance of aberrant crypt foci (ACF) precursors for colon cancer compared with rats receiving control water. They also found that resveratrol treatment reduced the appearance of large-sized ACF as well as increased the expression of *Bcl2*-associated X (*Bax*), a pro-apoptotic protein in precancerous cells. Other studies have used 1,2-dimethylhydrazine (DMH) to promote colon tumor formation in rats in order to study the chemopreventative effects of resveratrol. Sengottuvelan & Nalini exposed adult male Wistar rats to DMH once weekly for 15 weeks and then killed the rats 30 weeks after the initial DMH injection. The rats were then treated daily with vehicle or resveratrol (8 mg/kg BW; oral gavage): i) before and during DMH initiation (2 weeks before and throughout the 15 weeks DMH treatment), ii) after the final DMH treatment (2 days after the last DMH injection to the end of the study), and iii) from the initial DMH treatment until the end of the experiment. They found that all three resveratrol exposure protocols decreased tumor incidence and the number of ACF compared with vehicle-treated DMH rats (Sengottuvelan & Nalini 2006). This same group also looked at the markers of inflammation, cell proliferation, and apoptosis in intestinal mucosa sampled from rats injected with DMH, with or without a similar resveratrol supplementation protocol. Resveratrol reduced COX2 expression and activity, decreased ornithine decarboxylase which is highly expressed in cells during cell proliferation and tumor promotion, and increased the presence of cleaved capsase-3, indicating cellular apoptosis (Sengottuvelan *et al*. 2009). In 8-week-old Sprague–Dawley male rats, treatment with resveratrol by gavage over a 49-day period starting 7 days before tumor initiation (60 mg/kg BW) reduced the number of intestinal ACF as well as mucin-depleted foci (MDF) following DMH injection (Alfaras *et al*. 2010). MDF reduction is important because MDF foci are characterized by deregulated Wnt signaling (Yang *et al*. 2008), which is considered to be a major risk factor for colon cancer development (Moon *et al*. 2004, Sancho *et al*. 2004).

Other studies used a mouse model of spontaneous colon tumor formation. Adenomatous polyposis coli (Apc)^Min/+^ mice contain a germ line mutation in the tumor suppressor gene adenomatous polyposis and are predisposed to develop colon cancer (Wechter *et al*. 2000). Since spontaneous tumors in these mice are sensitive to COX inhibitors, resveratrol use to prevent tumor formation was of interest in this model (Jacoby *et al*. 1996, 2000). In one study, when 5-week-old male C57BL/6J-Apc^Min/+^ mice were treated with resveratrol for 7 weeks (0.01% in drinking water; daily intake calculated to be between 0.3 and 0.4 mg/mouse per day), resveratrol supplementation resulted in a 70% reduction in small intestinal tumors compared with vehicle-treated control animals. Markers for cell cycle progression and proliferation were evaluated in the intestinal mucosa and resveratrol decreased cyclins D1 and D2 (Schneider *et al*. 2001). Using 4-week-old C57BL/6J-Apc^Min/+^ mice, Sale *et al*. showed that resveratrol supplementation for 10–14 weeks in the diet (0.2% of diet; ∼240 mg/kg BW daily) reduced adenoma number in the colon and small intestine compared with control Apc^Min/+^ mice, but a lower dose did not significantly affect adenoma number (0.05% of diet; ∼60 mg/kg BW daily) (Sale *et al*. 2005). After 3 weeks on either the 0.05 or 0.2% resveratrol diet, levels of prostaglandin E2 (PGE2) in WT male C57BL/6J mice were reduced in the intestinal mucosa, suggesting decreased COX2 activity (Sale *et al*. 2005). Using C57BL/6J-Apc^Min/+^ mice, however, Ziegler *et al*. (2004) were unable to show that resveratrol supplementation prevented tumor formation. Specifically, the incidence of tumors or *Cox2* expression was no different between male mice given resveratrol (in diet; daily intake of 4, 20, or 90 mg/kg starting at ∼6 weeks of age for 7 weeks) vs control. Sale *et al*. only detected differences in adenoma load at a dose that was much higher than either Ziegler *et al*. (no effect) or Schneider *et al*. (positive effect). Age at which this mouse model is treated may be critical in preventing tumor formation; Sale *et al*. and Schneider *et al*. started resveratrol treatment at 4 and 5 weeks of age, respectively, while Ziegler *et al*. waited until 6 weeks of age to start the supplementation. Ziegler *et al*., however, did find a significant decrease in PGE2 levels in the tumors treated with resveratrol at 90 mg/kg BW, suggesting that resveratrol was having some effect on *Cox2* activity in the intestinal mucosa (Ziegler *et al*. 2004).

Finally, resveratrol in combination with other natural compounds may also be a viable option for the treatment of colon cancers. In a recent study, 7-week-old female ICR SCID mice were injected with HCT-116 (wt) cells to initiate colon cancer formation, and 15 days post cell injection after tumors had formed, they were treated daily by oral gavage with both 500 mg/kg of curcumin and 150 mg/kg of resveratrol for 3 weeks (Majumdar *et al*. 2009). The combination of curcumin and resveratrol were able to significantly inhibit colon cancer cell growth compared with tumor growth in vehicle control mice, and this was associated with increases in apoptotic cells in the treated mice. Both resveratrol and curcumin individually (at the same doses as the combination) provided significant, albeit lower levels of protection in this model as well (Majumdar *et al*. 2009). Table 2 summarizes the animal models used to investigate resveratrol and colorectal cancer.

### Liver cancer

Liver cancer, or hepatocellular carcinoma (HCC), is one of the most deadly forms of cancer, and its incidence has been increasing worldwide (Llovet *et al*. 2003). Risk factors for HCC include hepatitis, alcoholism, and high-fat diet consumption (El-Serag *et al*. 2006, Alter 2007). Animal models used to study the effects of resveratrol on hepatic tumors include transplantation of liver cancer cells into animal host and carcinogenic promotion of tumor formation. In adult male Wistar rats injected with AH-130 Yoshida ascites hepatoma cells, daily resveratrol treatment (1 mg/kg BW; i.p. injection) starting at cell implantation reduced the number of tumor cells after 7 days compared with untreated rats injected with the hepatoma cells. Though, it is important to point out that tumor volume was unchanged by resveratrol treatment (Carbo *et al*. 1999).

Multiple studies in rats have used diethylnitrosamine (DENA) injection followed by tumor promotion with phenobarbital to induce HCC in the animals. In one of these studies, Bishayee & Dhir (2009) gave resveratrol to adult female Sprague–Dawley rats (0.06, 0.12, or 0.36% diet; daily intake calculated to be 50, 100, or 300 mg/kg BW) starting 4 weeks before tumor initiation and continued for 16 weeks after initiation. Resveratrol supplementation at 100 and 300 mg/kg BW reduced the appearance and multiplicity of hepatocyte nodules at the end of the study compared with DENA-treated animals that did not receive resveratrol. Cellular architecture of the liver tissue was also improved by resveratrol treatment at 300 mg/kg BW. In all three doses of resveratrol treatment, there was a decrease in hepatic cellular proliferation as indicated by reduced expression of proliferating cell nuclear antigen (PCNA). In the livers of the two higher doses of resveratrol-treated animals, there were also significant increases in *Bax* expression and decreases in *Bcl2* expression, signifying facilitation of apoptosis by resveratrol. Furthermore, resveratrol at higher doses decreased lipid peroxidation and protein carbonyl content in livers compared with control DENA-injected rats, indicating that resveratrol might act as a free radical scavenger and decrease the incidence of tumor formation (Bishayee *et al*. 2010*a*). Resveratrol supplementation also caused an increase in hepatic expression of NFE2-related factor 2 (*Nrf2* (*Nfe212*); Bishayee *et al*. 2010*a*). Increased expression of *Nfr2*, a transcription factor involved in the expression of antioxidant genes, suggests that resveratrol may exert an antioxidant effect in the liver of DENA-injected animals. Lastly, in a dose-dependent manner, resveratrol reduced the expression of heat-shock protein 70 and COX2, as well as decreased DENA-induced translocation of NF-κB to the nucleus, suggesting that resveratrol is having an anti-inflammatory effect in this model (Bishayee *et al*. 2010*b*). In a separate study, the same group found that resveratrol reduced tumor multiplicity in a dose-dependent manner compared with control DENA-treated animals 14 weeks after tumor initiation. In this study, the lowest dose of resveratrol (50 mg/kg BW) also significantly decreased the nodule multiplicity (Luther *et al*. 2011). In a more recent study, Rajasekaran *et al*. (2011) have investigated the ability of resveratrol to prevent or treat HCC in 6- to 8-week-old male Wistar rats by treating animals daily with resveratrol (20 mg/kg BW; oral gavage) for either 15 days starting at the DENA injection or for 15 days after the development of HCC. In both the early and advance stages of HCC, resveratrol treatment increased the expression of apoptotic markers and decreased the expression of anti-apoptotic markers. Resveratrol treatment at both time points also reduced cell crowding and alteration in cellular architecture as well as decreased liver size compared with control rats treated with DENA (Rajasekaran *et al*. 2011). Lin *et al*. (2012) evaluated the effects of resveratrol treatment on the precancerous stage of liver carcinogenesis in 12-month-old male hepatitis B virus X protein (HBx) transgenic mice that spontaneously develop HCC at older ages. Resveratrol supplementation (0.024% diet; daily intake calculated to be 30 mg/kg BW) for 4 months significantly reduced the incidence of HCC by 5.3-fold and increased latency to tumor formation. The results from liver cancer models have been consistently positive, indicating a potential benefit for resveratrol in HCC prevention and/or therapy.

In addition to regulation of liver cancer function, resveratrol may also influence metastasis to the liver from other primary cancers. Salado *et al*. (2011) used B16 melanoma (B16M) cells to study the effects of resveratrol treatment on hepatic metastasis caused in large part by the production of proinflammatory cytokines. Six- to eight-week-old male C57BL/6J mice were given a daily oral dose of resveratrol (1 mg/kg BW) from the day of intrasplenic injection of B16M cells through 12 days after injection. Resveratrol treatments reduced hepatic metastasis volume and metastasis number compared with vehicle control mice given B16M cells. A summary of the methods and findings are given in Table 3.

### Pancreatic cancer

Risk for pancreatic cancer is linked to obesity, high-fat diet, and consumption of meat products (Olsen *et al*. 1989, Baghurst *et al*. 1991). Because so many pancreatic carcinomas are diagnosed at late, treatment-refractory stages, prognosis for patients with pancreatic cancer is generally poor. Clearly, there is a need for effective treatment alternatives for pancreatic cancer. A few researchers have looked at the *in vivo* effects of resveratrol on pancreatic cancer (Table 4). Oi *et al*. (2010) injected 6- to 8-week old Swiss nude mice with human pancreatic carcinoma cells (PaCa-2) to promote pancreatic tumor xenograft formation. Resveratrol was given five times a week by oral gavage (10 or 50 mg/kg BW) for 2 weeks before MIA PaCa-2 injection and then continued throughout the experiment until tumor volumes reached 1 cm^3^. Resveratrol reduced tumor size and number in a dose-dependent manner compared with animals dosed with vehicle control. Resveratrol also inhibited the activity of an inflammatory enzyme, leukotriene A_4_ hydrolase (Oi *et al*. 2010). In a similar study, Harikumar *et al*. (2010) injected MIA PaCa-2 cells into 4-week-old male mice and then resveratrol was given daily (40 mg/kg BW) for a 4 week duration starting 1 week after the cells were injected. They found that resveratrol treatment significantly decreased tumor growth compared with vehicle-treated mice. Combination treatment of resveratrol with gemcitabine further enhanced protection as well. In 4- to 6-week old BALB/c nude mice treated five times a week with resveratrol for 6 weeks (20, 40, or 60 mg/kg BW by gavage) starting 1 week after tumor cell injection, resveratrol reduced tumor growth caused by the injection of PANC-1 cells (human pancreatic carcinoma, epithelial-like cells) in a dose-dependent manner compared with control mice (Roy *et al*. 2011). Tumor tissues from resveratrol-treated mice also showed increased apoptosis and decreased proliferation compared with tumor tissue from vehicle-treated mice. This was accompanied by the inhibition of PI3K and Akt phosphorylation leading to an increase in the activation of the transcription factor Forkhead box O (FOXO). Activation of FOXO results in the expression of genes involved in cell-cycle arrest, indicating that resveratrol reduced tumor growth through its effects on the cell cycle (Roy *et al*. 2011). In 8-week-old Kras^*G12D*^ mice that spontaneously develop pancreatic tumors, resveratrol treatment over a 10-month period for five times a week (40 mg/kg BW by oral gavage) reduced pancreatic lesions compared with Kras^*G12D*^ that did not receive resveratrol treatment, indicating that resveratrol reduced spontaneous pancreatic tumors (Shankar *et al*. 2011). The use of all animal models of pancreatic cancer has not shown that resveratrol supplementation is beneficial, however. One study evaluated resveratrol treatment (0.001% in diet) in 6-week-old male Syrian hamsters during and after tumor initiation via *N*-nitrosobis(2-oxopropyl)amine injection (Kuroiwa *et al*. 2006). Resveratrol did not affect the formation of hyperplasias or adenocarcinomas in either treatment stage. Regardless, data at this point suggest that resveratrol either positively influences or does not significantly impact pancreatic cancer outcomes in rodent models.

### Prostate cancer

In men, prostate cancer is a leading cause of cancer-related death in USA (Siegel *et al*. 2013). Diet and lifestyle may play a major role in the development of prostate cancer (Wolk 2005), making a supplement such as resveratrol a promising candidate for prostate cancer chemoprevention. Relatively few *in vivo* studies, however, have been conducted that investigate the effects of resveratrol on prostate cancer prevention and treatment. Two studies have used rodent models of spontaneous prostate tumor formation to investigate resveratrol's protective abilities: the transgenic adenocarcinoma of mouse prostate (TRAMP) and the transgenic rat for adenocarcinoma of prostate (TRAP) models (Harper *et al*. 2007, Seeni *et al*. 2008). Three additional studies have used xenograft models to study resveratrol's potential effects (Seeni *et al*. 2008, Wang *et al*. 2008, Dias *et al*. 2013).

Harper *et al*. (2007) administered resveratrol (0.0625% in diet) to 5-week-old TRAMP mice daily for 23 weeks and then observed tumor formation in the urogenital tract of the mice. Resveratrol-fed mice had a significantly reduced percentage of Grade 6, poorly differentiated tumors compared with control diet-fed mice. Grade 4 lesions were more common in resveratrol-treated mice than control diet-fed, indicating that resveratrol slowed down tumor progression to this stage (Harper *et al*. 2007). There were, however, no differences in tumor numbers per animal, tumor weight, latency to tumor formation, or metastases between resveratrol treated and control animals (Harper *et al*. 2007). Further, at 12 weeks of age after 7 weeks on the resveratrol diet, TRAMP mice showed reduced cellular proliferation in the dorsolateral and ventral prostate compared with control diet mice (Harper *et al*. 2007).

When resveratrol was administered daily to TRAP rats for 7 weeks (0.005, 0.01, or 0.02% in drinking water), neoplastic lesion development was significantly reduced in the ventral and lateral lobes of the prostate compared with control-treated TRAP rats (Seeni *et al*. 2008). There were, however, no significant differences in adenocarcinoma incidence between resveratrol-treated and control TRAP rats in either the ventral or lateral prostate (Seeni *et al*. 2008). Protein expression of androgen receptor (AR) was also measured in the prostate of TRAP rats; resveratrol treatment at all three doses significantly decreased AR expression in the ventral prostate compared with control animals, suggesting a possible mechanism through which resveratrol may have chemopreventative effects (Seeni *et al*. 2008). Within this same paper, Seeni *et al*. injected rat prostate cancer cells (PLS30 cells) into male athymic CD-1 nude mice at 6 weeks of age. Mice were then treated with or without resveratrol (0.01 or 0.02% in drinking water) from 1 week after the injection until they were killed at 6 weeks after the injection. Tumor volume and metastatic foci in the lungs were measured; neither resveratrol dose significantly affected either parameter compared with those mice not treated with resveratrol. Seeni *et al*. (2008) hypothesized that this was possibly due to the lack of AR protein in the PLS30 cells.

Two other studies have focused on the effects of resveratrol in human prostate cancer cells xenograft models. In both, androgen responsive-LNCaP human prostate cancer cells were injected s.c. into male mice following pre-treatment with resveratrol. Wang *et al*. (2008) administered resveratrol to 5-week-old BALB/cAnNCr-*nu/nu* mice (0.005 or 0.01% in diet, daily) for 2 weeks before cell injection through 7 weeks after injection. After injection, animals were palpitated for tumor growth weekly; resveratrol at both doses in the diet significantly delayed tumor growth by 3 weeks (both doses) and 4 weeks (higher dose only) post injection compared with control diet mice; however, by 7 weeks, there were no differences in tumor volume (Wang *et al*. 2008). No differences in cell proliferation, measured by PCNA, were found between the resveratrol-treated and untreated groups. Interestingly, the resveratrol-treated animals showed significantly lower levels of apoptosis, measured by ApopTag, and there was increased microvessel formation (angiogenesis), measured by platelet/endothelial cell adhesion molecule 1 staining, in the higher resveratrol dose mice compared with control-fed mice (Wang *et al*. 2008). Deceased apoptosis and increased angiogenesis could lead to long-term complications and worse outcomes. A separate study used similar tumor cells in 6- to 7-week-old Fox n1^nu^ mice (Dias *et al*. 2013). Mice were treated with 50 mg/kg of resveratrol via oral gavage every other day for 2 weeks before tumor cell injection through 5 weeks after injection. Resveratrol significantly decreased tumor formation and progression, as assessed by caliper measurements, compared with control mice that did not receive resveratrol. Resveratrol also caused a decrease in serum interleukin 6 (IL6; Dias *et al*. 2013). As part of the same experiment, Dias *et al*. (2013) also studied the effects of two resveratrol analogs that could have better bioavailability (trimethoxy-resveratrol and piceatannol) and found that both decreased tumor volume and IL6 in mouse serum. These compounds should be tested in additional studies. Data from these models suggest that resveratrol may have some positive impacts on prostate tumor formation and progression, but it may have some unwanted effects on angiogenesis around the tumors. Additional work needs to be done in this area. A summary of the prostate cancer studies is provided in Table 5.

## Discussion

Although there is some *in vivo* evidence for the use of resveratrol as a chemopreventative agent, there is still much more research that needs to be done on tumor induction methods and dose selection as well as age- and sex-specific effects of resveratrol supplementation. Tables 1, 2, 3, 4 and 5 highlight the differences in the *in vivo* models used, including cancer models, methods of tumor initiation, strain, species, sex, method/timing of resveratrol administration, and dose of resveratrol. The tables show that there is no consistent technique used for resveratrol administration. Resveratrol delivery (large single daily dose by oral gavage or injection vs small doses throughout the day/night when resveratrol is provided in food or drinking water) could have critical effects on cancer outcomes due to the quick absorption and metabolism of resveratrol. Furthermore, many papers did not measure or report circulating resveratrol levels which makes comparison across strains, species, and studies nearly impossible. The source (purified from Japanese knotweed, chemically synthesized, etc.) and purity of resveratrol were not reported in all papers and could also play a role in inconsistent effects observed in the studies. It is also important to take into account the fact that doses in animals generally cannot be directly translated to humans, for example, it may be important to normalize dosing to body surface area as opposed to BW (Reagan-Shaw *et al*. 2008).

There is little evidence from animal or human studies that resveratrol can serve as a viable treatment option once tumors are already formed, so it is not likely that resveratrol can be used as an alternative for the traditional forms of cancer treatment in the near future. Further, resveratrol supplementation had no effect on spontaneous neoplasia formation in WT C57BL/6 male mice fed resveratrol in their diet (0.01 or 0.04%) from 12 months of age through the remainder of their lives (Pearson *et al*. 2008). Also, resveratrol does not appear to target the cellular structures involved in proliferation such as microtubules or nucleotide synthetic enzymes that many of the traditional chemotherapeutics target; therefore resveratrol is unlikely to be efficacious as a primary anti-cancer agent. Rather, resveratrol appears to maintain cellular homeostasis in part by protecting cells against oxidative injury and other cancer-causing perturbations. The addition of resveratrol to standard chemotherapeutic regimens may therefore be helpful in preventing the development of secondary malignancies that result from mutagenic effects of chemotherapy and radiotherapy (Kinghorn *et al*. 2004, Aziz *et al*. 2005, Le Corre *et al*. 2005, Lee & Lee 2006, Khan *et al*. 2008, Kundu & Surh 2008, Dennis *et al*. 2009, Seehusen *et al*. 2010, Newhauser & Durante 2011, Szekeres *et al*. 2011). Resveratrol may also help to prevent other long-term morbidities associated with anti-cancer therapy, such as cardiac myocyte toxicity and subsequent heart failure from exposure to anthracyclines such as doxorubicin (Tatlidede *et al*. 2009). Thus, although resveratrol is not likely to be a primary treatment for cancer, in addition to its potential role in primary cancer prevention by reducing carcinogenesis for primary malignancies, it may have a role in the prevention of secondary malignancies and/or other toxic effects of traditional chemotherapeutic agents. As a result, it is important to emphasize further resveratrol supplementation as a way to prevent the development of cancer and disease and as a supplement used in conjugation with traditional chemotherapeutics.

After oral administration, *trans*-resveratrol is quickly conjugated into glucuronides and sulfates (Andlauer *et al*. 2000, De Santi *et al*. 2000, Soleas *et al*. 2001, Yu *et al*. 2002). Because the bioavailability of *trans*-resveratrol after oral administration is low (Wenzel & Somoza 2005), researchers have recently started to investigate the effects of resveratrol derivatives with higher bioavailability (Szekeres *et al*. 2011, Dias *et al*. 2013). Also, some of the efforts to improve resveratrol's bioavailability have focused on combination therapy with other compounds that may prevent or delay conjugation of resveratrol. Piperine, a compound found in black pepper, can inhibit glucuronidation (Reen *et al*. 1993, Shoba *et al*. 1998). In mice, piperine significantly increased the serum levels of resveratrol after oral administration of both compounds (Johnson *et al*. 2011). Other studies have focused on the synergistic effects of resveratrol and other naturally occurring compounds such as melatonin, tea polyphenols, and quercetin on cancer models; for a thorough review of these studies see Singh *et al*. (2013).

Epidemiological studies have found strong correlations between obesity and certain types of cancers including breast, endometrial, colorectal, pancreatic, and HCCs (Moller *et al*. 1994, Galanis *et al*. 1998, Silverman *et al*. 1998, Gapstur *et al*. 2000, Trentham-Dietz *et al*. 2000, Vainio *et al*. 2002, Calle *et al*. 2003). Excessive amounts of body fat can cause changes in hormone and protein levels that result in cellular deregulation, and therefore possibly cancer. Therefore, targeting obesity may be a way to prevent and/or lower risk of cancer development. Resveratrol is an important molecule to consider in this area of cancer prevention since it has been shown to have many positive effects on animal models of obesity and high-fat diet. In animal studies, resveratrol was found to prolong survival and decrease fat mass in mice fed a high-fat diet (Baur *et al*. 2006, Lagouge *et al*. 2006). Resveratrol treatment also decreased hyperglycemia in a model of diet-induced diabetes as well as improved insulin sensitivity when insulin resistance has developed due to increased fat mass (Ramadori *et al*. 2009, Kang *et al*. 2012). In some initial clinical trials, resveratrol supplementation improved glucose regulation in aged subjects with impaired glucose tolerance and improved homeostatic model assessment index in obese men (Timmers *et al*. 2011, Crandall *et al*. 2012). Also, in two separate studies looking at resveratrol supplementation in type 2 diabetics, resveratrol improved insulin sensitivity and HbA1c measurements (Brasnyo *et al*. 2011, Bhatt *et al*. 2012). Other groups were unable to show improvements in insulin sensitivity or glucose regulation with resveratrol treatment. In both normal weight and obese subjects, resveratrol use was unable to improve insulin sensitivity and glucose uptake into tissues when measured by hyperinsulinemic–euglycemic clamp (Yoshino *et al*. 2012, Poulsen *et al*. 2013). Similar to the animal studies looking at resveratrol and cancer, differences in the amount and length of resveratrol treatment could be a factor in differences observed between studies. However, there is some promising evidence that resveratrol can improve metabolic outcomes and could have a major impact on overall health, including decreasing cancer risk.

## Conclusion

Research has shown that resveratrol supplementation could potentially have many positive health benefits including decreased cancer risk. Yet, there are limited clinical trials with small sample sizes, and animal models have had mixed results. There is a need for more extensive and consistent studies in animal models. Little evidence exists that resveratrol can be used effectively to treat preexisting tumors (that were not implanted cells); therefore the most promising use of resveratrol is most likely as a cancer preventative agent. Determining the efficacy and appropriateness for resveratrol as a cancer preventive or anti-cancer agent is likely to be an area of emphasis for future research studies and clinical trials. Resveratrol may impact certain tumor types more so than others, based on its proposed mechanisms of action and different oncogenic pathways being tumor-specific. In addition, much work needs to be done on optimizing the bioavailability of the drug and determining its pharmacokinetic, pharmacodynamics, and safety profile in different patient populations (e.g. adults vs pregnant women vs children).

## Figures and Tables

**Table 1:** Breast cancer

**References**	**Strain/species**	**Sex**	**Age**^a^	**Tumor model**	**Resveratrol dose and administration**	**Effect on tumorigenesis**^b^
Bhat *et al*. (2001)	Sprague–Dawley rats	F	42 days	NMU	I.g.; 10 or 100 mg/kg BW; 5×/week; 7 days before initiation – 120 days after	Positive^c^
Banerjee *et al*. (2002)	Sprague–Dawley rats	F	45 days	DMBA	0.001% in diet; 100 μg/rat daily; 7 days before initiation – 120 days after initiation	Positive
Bove *et al*. (2002)	BALB/c mice	F	17 weeks	4T1 cells	I.p.; 1, 3, or 5 mg/kg BW; daily; 23 days started at injection	Unchanged
Sato *et al*. (2003)	Sprague–Dawley rats	F	15 days	NMU	S.c.; 10 or 100 mg/kg BW; daily for 5 days; from 30 to 34 days before initiation	Negative^c^
Provinciali *et al*. (2005)	HER2/*neu* mice	F	20 weeks	Spontaneous tumors	0.0001% in drinking water; 4 μg/mouse daily; for 11 weeks	Positive
Garvin *et al*. (2006)	Nude mice	F	6–8 weeks	MDA-MB-231 (ERα(−), ERβ(+)) cells	I.p.; 25 mg/kg BW; daily; for 3 weeks after tumor size reached 40 mm^3^	Positive
Whitsett *et al*. (2006)	Sprague–Dawley rats	F	0 days	DMBA	0.1% in diet; daily; 50 days before initiation – 18 weeks after initiation	Positive
Chatterjee *et al*. (2011)	Sprague–Dawley rats	F	5 weeks	DMBA	0.001% in diet; 100 μg/rat daily; 2 weeks before initiation – 24 weeks after initiation	Positive
Castillo-Pichardo *et al*. (2013)	SCID mice	F	5–6 weeks	MDA-MB-231 (ERα(−), ERβ(+)) cells	Gavage; 0.5, 5, or 50 mg/kg BW; 5×/week; 7 days after injection for 108 days	Negative
Castillo-Pichardo *et al*. (2013)	Nude mice	F	5–6 weeks	MDA-MB-435 (ER(−)) cells	Gavage; 0.5, 5, or 50 mg/kg BW; 5×/week; 7 days after injection for 44 days	Negative

BW, body weight; DMBA, 7,12-dimethylbenz(*a*)anthracene; ER, estrogen receptor; F, female; i.g., intragastric intubation; NMU, *N*-nitroso-*N*-methylurea; SCID, severe combined immunodeficiency.

a Age in table indicates age of animal when study was started; either when tumors were initiated or when resveratrol was administered, depending on study design.

b Review authors' interpretation of paper results with a focus on tumor outcomes.

c Lower dose did not significantly affect outcomes.

**Table 2:** Colorectal cancer

**References**	**Strain/species**	**Sex**	**Age**^a^	**Tumor model**	**Resveratrol dose and administration**	**Effect on tumorigenesis**^b^
Tessitore *et al*. (2000)	F344 rats	M	8 weeks	AOM	In drinking water; 200 μg/kg BW daily; 10 days before initiation, continued for 100 days	Positive
Schneider *et al*. (2001)	APC^Min^/+ mice	M	5 weeks	Spontaneous tumors	0.01% in drinking water; between 0.3 and 0.4 mg/mouse daily for 7 weeks	Positive
Ziegler *et al*. (2004)	APC^Min^/+ mice	M	43 days	Spontaneous tumors	In diet; 4, 20, or 90 mg/kg BW daily for 7 weeks	Unchanged
Sale *et al*. (2005)	APC^Min^/+ mice	M	4 weeks	Spontaneous tumors	0.05 or 0.2% in diet; 60 or 240 mg/kg BW daily for 10–14 weeks	Positive^c^
Sengottuvelan & Nalini (2006)	Wistar rats	M	Adult	DMH	I.g.; 8 mg/kg BW; daily; 2 weeks before first DMH – final DMH^d^	Positive
Sengottuvelan & Nalini (2006)	Wistar rats	M	Adult	DMH	I.g.; 8 mg/kg BW; daily; 2 days after final DMH – 15 weeks after final DMH^d^	Positive
Sengottuvelan & Nalini (2006)	Wistar rats	M	Adult	DMH	I.g.; 8 mg/kg BW; daily; on day of first DMH – 30 weeks after^d^	Positive
Majumdar *et al*. (2009)	SCID mice	F	7 weeks	HCT-116 (wt) cells	Gavage; 150 mg/kg BW; daily; 15 days after injection for 3 weeks	Positive
Alfaras *et al*. (2010)	Sprague–Dawley rats	M	8 weeks	DMH	Gavage; 60 mg/kg BW; daily; 7 days before initiation for 49 days	Positive

AOM, azoxymethane; BW, body weight; DMH, 1,2-dimethylhydrazine; i.g., intragastric intubation; M, male; SCID, severe combined immunodeficiency.

a Age in table indicates age of animal when study was started; either when tumors were initiated or when resveratrol was administered, depending on study design.

b Review authors' interpretation of paper results with a focus on tumor outcomes.

c Unchanged for lower dose.

d DMH was given once weekly for 15 weeks, and then the rats were killed 15 weeks after the last DMH injection (30 weeks after initial DMH exposure).

**Table 3:** Liver cancer

**References**	**Strain/species**	**Sex**	**Age**^a^	**Tumor model**	**Resveratrol dose and administration**	**Effect on tumorigenesis**^b^
Carbo *et al*. (1999)	Wistar rats	M	Adult^c^	AH-130 cells	I.p.; 1 mg/kg BW; daily; 7 days starting at injection	Positive
Bishayee & Dhir (2009)	Sprague–Dawley rats	F	31–37 days^d^	DENA+PB	0.06, 0.12, or 0.36% in diet; 50, 100, or 300 mg/kg BW daily; 4 weeks before initiation – 16 weeks after	Positive^e^
Luther *et al*. (2011)	Sprague–Dawley rats	F	31–37 days	DENA+PB	0.06, 0.12, or 0.36% in diet; 50, 100, or 300 mg/kg BW daily; 4 weeks before initiation – 14 weeks after	Positive
Rajasekaran *et al*. (2011)	Wistar rats	M	6–8 weeks	DENA+PB	Gavage; 20 mg/kg BW; daily; on day of initiation – 15 days after	Positive
Rajasekaran *et al*. (2011)	Wistar rats	M	6–8 weeks	DENA+PB	Gavage; 20 mg/kg BW; daily; for 15 days from 17 to 18 weeks after initiation	Positive
Salado *et al*. (2011)	C57BL/6J mice	M	6–8 weeks	B16M cells metastasis	I.g.; 1 mg/kg BW; daily; day of injection – 12 days after	Positive
Lin *et al*. (2012)	HBx mice	M	12 months	Spontaneous tumors	0.024% in diet; 30 mg/kg BW daily; for 4 months	Positive

BW, body weight; DENA, diethylnitrosamine; F, female; HBx, hepatitis B virus X protein; i.g., intragastric intubation; M, male; PB, phenobarbital.

a Age in table indicates age of animal when study was started; either when tumors were initiated or when resveratrol was administered, depending on study design.

b Review authors' interpretation of paper results with a focus on tumor outcomes.

c Age was not given but the rats were ∼100 g.

d Age was not given but the rats were the same weight (65–85 g) as the group's next paper (Luther *et al*. 2011).

e Unchanged for lowest dose.

**Table 4:** Pancreatic cancer

**References**	**Strain/species**	**Sex**	**Age**^a^	**Tumor model**	**Resveratrol dose and administration**	**Effect on tumorigenesis**^b^
Kuroiwa *et al*. (2006)	Syrian hamsters	M	6 weeks	BOP	0.001% in diet; daily; 1 week before initiation for 3 weeks	Unchanged
Kuroiwa *et al*. (2006)	Syrian hamsters	M	6 weeks	BOP	0.001% in diet; daily; 1 week after initial BOP injection for 14 weeks	Unchanged
Harikumar *et al*. (2010)	Nude mice	M	4 weeks	MIA PaCa-2 cells	Gavage; 40 mg/kg BW; daily; 1 week after injection for 4 weeks	Positive
Oi *et al*. (2010)	Nude mice	–	6–8 weeks	MIA PaCa-2 cells	Gavage; 10 or 50 mg/kg BW; 5×/week; 2 weeks before injection – tumors reaching 1 cm^3^ volume	Positive^c^
Roy *et al*. (2011)	Nude mice	–	4–6 weeks	PANC-1 cells	Gavage; 20, 40, or 60 mg/kg BW; 5×/week, 1 week after injection for 6 weeks	Positive
Shankar *et al*. (2011)	Kras^G12^D mice	–	8 weeks	Spontaneous tumors	Gavage; 40 mg/kg BW; 5×/week; for ∼10 months	Positive

BOP, *N*-nitrosobis(2-oxopropyl)amine); BW, body weight; M, male.

a Age in table indicates age of animal when study was started; either when tumors were initiated or when resveratrol was administered, depending on study design.

b Review authors' interpretation of paper results with a focus on tumor outcomes.

c The protection was dose dependent, but the lower dose was not significantly improved compared to vehicle control.

**Table 5:** Prostate cancer

**References**	**Strain/species**	**Sex**	**Age**^a^	**Tumor model**	**Resveratrol dose and administration**	**Effect on tumorigenesis**^b^
Harper *et al*. (2007)	TRAMP mice	M	5 weeks	Spontaneous tumors	0.0625% in diet; daily for 23 weeks	Positive
Seeni *et al*. (2008)	TRAP rats	M	3 weeks	Spontaneous tumors	0.005, 0.01, or 0.02% in drinking water; daily for 7 weeks	Positive
Seeni *et al*. (2008)	Nude mice	M	6 weeks	PLS30 cells	0.01 or 0.02% daily in drinking water; 1 week after cell injection – 6 weeks after	Unchanged
Wang *et al*. (2008)	Nude mice	M	5 weeks	LNCaP cells	0.005 or 0.01% in diet; daily; 2 weeks before cell injection – 7 weeks after	Unchanged^c^
Dias *et al*. (2013)	Nude mice	M	6–7 weeks	LNCaP cells	Gavage; 50 mg/kg BW; every other day; 2 weeks prior to cell injection – 5 weeks after	Positive

BW, body weight; M, male; TRAP, transgenic rats for adenocarcinoma of prostate; TRAMP, transgenic adenocarcinoma of mouse prostate.

a Age in table indicates age of animal when study was started; either when tumors were initiated or when resveratrol was administered, depending on study design.

b Review authors' interpretation of paper results with a focus on tumor outcomes.

c Resveratrol delayed tumor growth at 3 weeks (both doses) and 4 weeks (higher dose only) post injection compared with mice on control diet, but there were no differences in tumor size after 7 weeks at the end of the study.
